# Structure Prediction, Molecular Dynamics Simulation and Docking Studies of D-Specific Dehalogenase from *Rhizobium* sp. RC1

**DOI:** 10.3390/ijms131215724

**Published:** 2012-11-26

**Authors:** Ismaila Yada Sudi, Ee Lin Wong, Kwee Hong Joyce-Tan, Mohd Shahir Shamsir, Haryati Jamaluddin, Fahrul Huyop

**Affiliations:** 1 Faculty of Biosciences and Medical Engineering, Universiti Teknologi Malaysia, UTM Skudai, 81310 Johor Bahru, Malaysia; E-Mails: yada280@ymail.com (I.Y.S.); elwong0101@gmail.com (E.L.W.); shahirshamsir@gmail.com (M.S.S.); haryati@fbb.utm.my (H.J.); 2 School of Bioscience and Technology, Faculty Science and Technology, Universiti Kebangsaan Malaysia, 43600 UKM Bangi, Selangor, Malaysia; E-Mail: joyssstan0202@gmail.com

**Keywords:** D-stereospecific dehalogenase, *Rhizobium* sp. RC1, DehD, interacting residues, docking, molecular dynamics simulation

## Abstract

Currently, there is no three-dimensional structure of D-specific dehalogenase (DehD) in the protein database. We modeled DehD using *ab initio* technique, performed molecular dynamics (MD) simulation and docking of D-2-chloropropionate (D-2CP), D-2-bromopropionate (D-2BP), monochloroacetate (MCA), monobromoacetate (MBA), 2,2-dichloropropionate (2,2-DCP), D,L-2,3-dichloropropionate (D,L-2,3-DCP), and 3-chloropropionate (3-CP) into the DehD active site. The sequences of DehD and D-2-haloacid dehalogenase (HadD) from *Pseudomonas putida* AJ1 have 15% sequence similarity. The model had 80% of the amino acid residues in the most favored region when compared to the crystal structure of DehI from *Pseudomonas putida* PP3. Docking analysis revealed that Arg107, Arg134 and Tyr135 interacted with D-2CP, and Glu20 activated the water molecule for hydrolytic dehalogenation. Single residue substitutions at 25–30 °C showed that polar residues of DehD were stable when substituted with nonpolar residues and showed a decrease in activity within the same temperature range. The molecular dynamics simulation of DehD and its variants showed that in R134A variant, Arg107 interacted with D-2CP, while in Y135A, Gln221 and Arg231 interacted with D-2CP. It is our emphatic belief that the new model will be useful for the rational design of DehDs with enhanced potentials.

## 1. Introduction

Haloalkanoic acids are widely used in the manufacturing and chemical industries as herbicides, insecticides and organic solvents, thereby introducing recalcitrant, toxic and transformed xenobiotic metabolites into the biosphere. D-2-specific dehalogenase (DehD) is a member of group I dehalogenases that catalyzes the hydrolytic dehalogenation of D-specific, low molecular weight halogenated compounds (environmental contaminants that result from agricultural activities, sewage treatment and the manufacturing and chemical industries) at the α-carbon position using a hydrolytic S_N_2 substitution reaction, resulting in the inversion of the substrate’s configuration. The mechanistic reaction involves nucleophilic attack at the α-carbon of the haloalkanoic acid by an Asp residue to form an ester intermediate and results in the release of halide ion. An activated water molecule then attacks the Asp residue at the γ-carbon to break the enzyme–product complex ester bond [1–8].

DehD was initially isolated from *Rhizobium* sp. by Berry and colleagues [9] (*Rhizobia* are bacteria that live in the soil or in the root nodules of legumes important to nitrogen fixation) and HadD was isolated from *Pseudomonas putida* AJI [10–12]. Both enzymes catalyze the dehalogenation of D-substrates, unlike non-stereospecific dehalogenase (DehE), which processes both D- and L-substrates [13–14]. DehD processes D-2CP to yield L-lactic acid, a process that has wide applications in agriculture, the manufacturing industries, and medical biotechnology. Biochemical pesticides made from L-lactic acid are used on food and animal feed as traps for mosquitoes. In recent times, L-lactate has been employed as a plant growth regulator, and as L-lactide (poly-L-lactic acid), in the synthesis of bicyclic and tricyclic lactide derivatives, as well as in biodegradable devices and the treatment of atrophic acne scarring. Similarly, poly-L-lactic acid has been used in the fabrication of biocompatible and biodegradable foams and has a potential use in tissue engineering. Recently, biodegradable poly-L-lactic acid stents have been used for drug delivery and in soft tissue augmentation during HIV-associated facial lipoatrophy [15–23]. It has been reported that development of dehalogenating enzymes like haloalkanoic acid dehalogenases (EC. 3.8.1.2), hydrogen-halide lyases (EC. 4.5.1) and haloalkane dehalogenases (EC. 3.8.1) for industrial biocatalysis has been limited [7]. Hydrolytic dehalogenases are utilized for commercial and industrial purposes though still at a developmental stage. The use of dehalogenase in industries is important for the manufacture of chiral intermediates, recycling of chlorinated byproducts from chemical manufacturing and in selective treatment of process waste streams [7]. HadD from *Pseudomanas putida* was employed for production of L-2CP as a chiral feedstock chemical for herbicide production (ICI patent no. 179603) and pharmaceutical products [7,24]. DehD from *Rhizobium* sp. RC1 could be exploited as a potential target enzyme for industrial, pharmaceutical and other biotechnological applications.

DehD has been partially purified [6,25,26]. It consists of 265 amino acid residues. It has been shown to act on D-2-chloropropionate (D-2CP), D-2-bromopropionate (D-2BP), monochloroacetate (MCA), monobromoacetae (MBA), D,L-2,3-dichloropropionate (D,L-2,3-DCP), and does not act on 2,2-dichlopropropionate (2,2-DCP), dichloroacetate (DCA), trichloroacetate (TCA), dibromoacetate (DBA), tribromoacetate (TBA) and 3-chloropropionate (3-CP) (a β-haloalkanoic acid). Non-stereospecific dehalogenase (DehE) and L-specific dehalogenase (DehL) could not act on 3-CP, as well [26–30].

The crystal structure of a representative member of group I (DehI) from *Pseudomonas putida* PP3 has been reported [13]. It consists of 297 amino acid residues, has a molecular mass of 32.7 kD, and is a highly α-helical homodimer composed of a repeated motif that has no structural homolog to DehD [13,26,28]. However, this DehI has insignificant amino acid sequence homology to DehD. Therefore, it is pertinent to explore the structure and key catalytic residues of DehD characterizing its function. The computational prediction of the three-dimensional (3D) structure of DehD could be of benefit in predicting its function, particularly because structure is more conserved than sequence between homologous proteins [31,32]. To the best of the authors’ knowledge, the crystallographic structure of DehD has not been reported in the protein database, and its catalytic residues have not yet been elucidated [28]. The present study used the *ab initio* modeling technique to determine the three-dimensional structure for DehD from its primary sequence and to model the docking of D-2CP into the protein’s active site in order to understand the key catalytic residues involved. We have subsequently predicted the structure of DehD and deduced the roles of its interacting residues, and we propose these amino acid residues as targets for site-directed mutagenesis so that the capabilities of this protein can be enhanced.

## 2. Results and Discussion

### 2.1. The Primary and Secondary Structure of DehD

The primary structure of DehD (accession number CAA63793.1 and molecular formula of C_1289_H_2081_N_391_O_370_S_10_) is composed of 265 amino acid residues, encoded by 798 nucleotides [26]. In contrast, DehI from *Pseudomonas* sp. strain PP3 contains 296 amino acid residues with a molecular weight of 32.7 kDa [13] and HadD, from the biochemically equivalent *Pseudomonas putida* AJ1, which consists of 301 amino acid residues, with a molecular weight of approximately 135 kDa [11].

The amino acid sequence of DehD was not observed to be similar to the previously crystallized representative member of DehI, from the *Pseudomonas putida* strain PP3. The similarity of DehD to other proteins was used to assess the statistical significance of the global alignment to generate sequence pairs of the appropriate length and composition by chance [33,34].

A protein blast search (BLASTp) indicated that DehD was only 15% identical to the conserved sequences from DehI and HadD [13,35]. This observation suggests that these proteins may not have the same functional residues responsible for the dehalogenation process. The amino acid sequence alignment patterns provided information on the conservation of core residues, such as the hydrophobic core and regions that are important for protein function [8,36]. The amino acid sequences of DehD and HadD showed a sequence identity of 15% between these two enzymes (see supplementary material), rather than 27%, that was reported previously [28]. This observed low similarity is an indication that these dehalogenases do not share the same key catalytic residues stereospecific to D-substrates.

**Figure 1:**
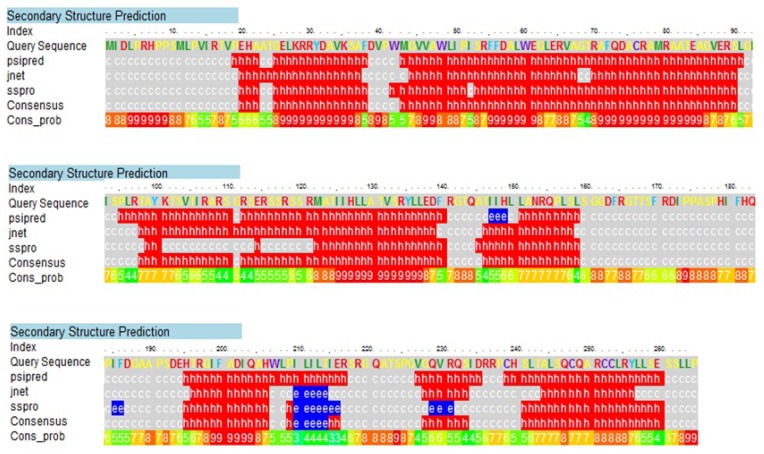
Secondary structural elements (SSEs) of DehD. Letters h stands for helix, c for coil and e for strand.

**Figure 2:**
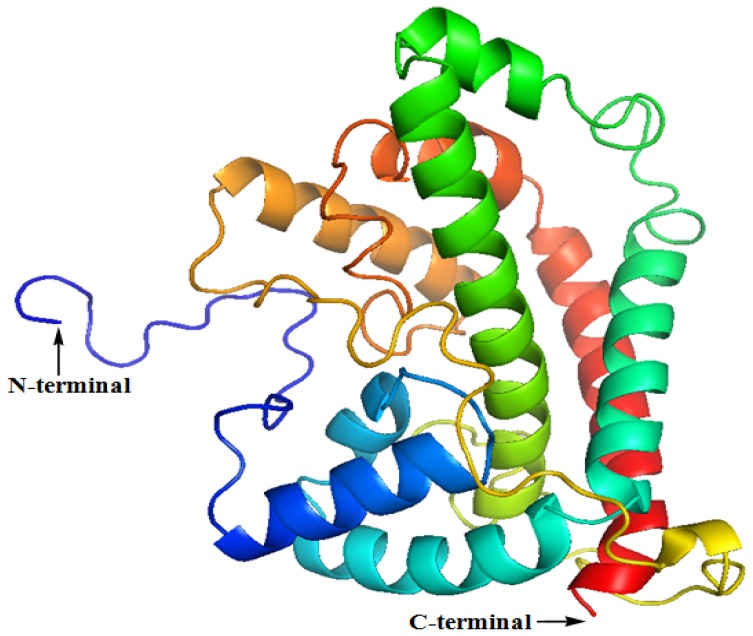
Secondary structure of DehD. *N*-terminal is shown in blue and C-terminal in red.

The secondary structure of the DehD was determined using a Phyre server (Figure 1, Table 1) and visualized using PyMol (Figure 2). The structure was predominantly α-helical. The *N*-terminus consists of 24 residues, primarily in the loop region, whereas the *C*-terminus had only one residue in the loop region. The D-2-specific dehalogenase (DehD) was found to be predominantly made of α-helices and coil–coils with highly curved bends and was without strands [37–39]. The server compares the result from three different program sources: psipred, jnet and sspro (Figure 1). All programs established that DehD structure is composed of 52% α-helices, 17% turns and 13% bends, and there was no consensus on the presence of strands that resulted in an insignificant strand in DehD. Two helical steps were observed at Thr167-Gly169 and at Gly220-Ile223. It also has two helical breaks at Pro51-Ile52 and at Gly111-Ser113. The highest strand consensus probability of six was located at His149 by psipred and located at Glu229 and Val230 by sspro. The lowest strand consensus probability of three was located at Ile210, Ile215 and Glu216 by all the programs, and a consensus of four at Leu211-Thr214. The structure was highly α-helical consisting of a total of 173 (65.28%) residues within eight helices of various lengths. The longest helix was Gly228–Leu264 (37 residues), followed by Gly5–Ala37 (33 residues), then Gln114–Arg141 (28 residues), then Arg70–Val87 (18 residues), Gln194–Ile210 (17 residues), Ser53–Gly68 (16 residues) and helical steps at Thr167–Gly169 and Gly220–Ile223 (three and four residues, respectively). The loops consisting of 112 (42.26%) amino acid residues were predominantly found at Met1–Thr24, Phe38–Trp42, Glu88–Lys101, Gly111–Ser113, Gly142–Gly166, Phe170–Asp193, Leu211–Arg219, and Ser224–Val227. The longest loop was Gly142–Gly166 (25 amino acid residues) followed by Met1–Thr24 and Phe170–Asp193 (24 amino acid residues each) and Glu88–Lys101 (14 amino acid residues). The shortest loop was Phe38–Trp42 (consist of five amino acid residues) and Leu211–Arg219 (nine amino acids residues). The strands were limited to the following places: Ile147–His149 (psipred), Ile184–Phe185 (sspro), Ile210–Thr14 (consensus among the three servers-psipred, jnet and sspro)/Ile215–Glu216 (sspro) and Gln229–Arg231 (sspro) (Figure 1). There was no consensus between these three servers on the presence of a strand in DehD.

### 2.2. The Three-Dimensional (3D) Structure of DehD

**Figure 3:**
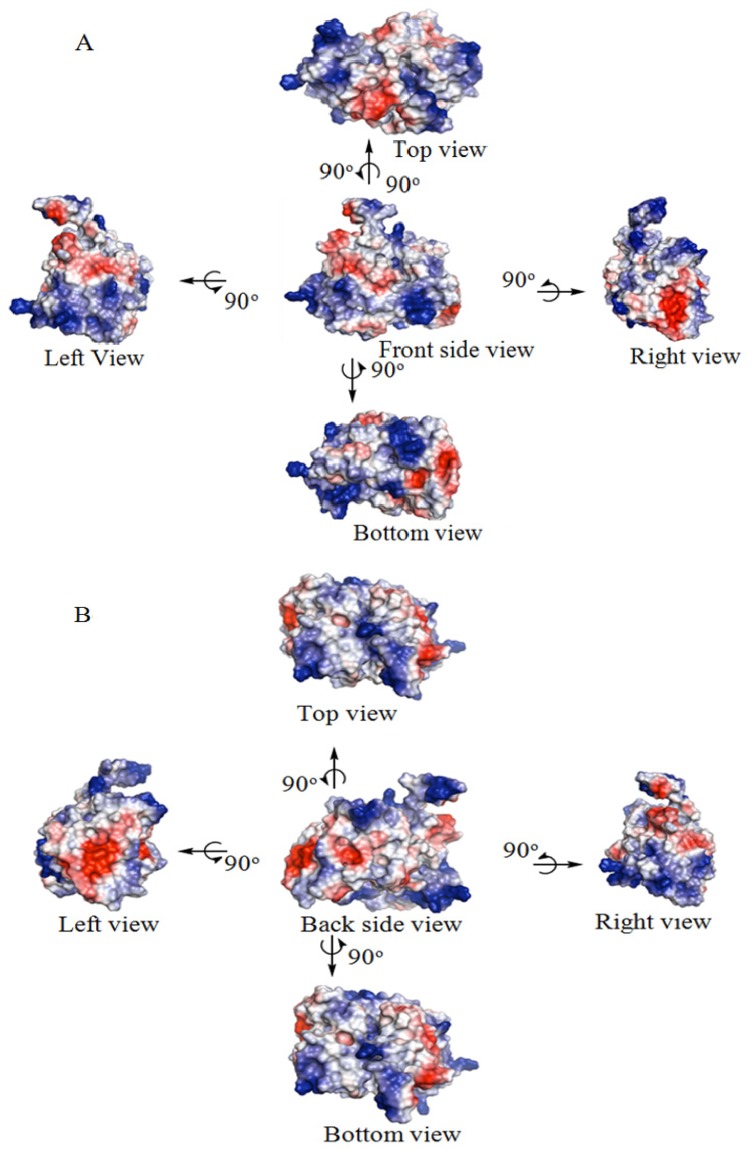
DehD Structure showing the accessible surface colored according to electrostatic potential (ESP). (**A**) Shows graphical rotation of front side view of the accessible surface. (**B**) Shows graphical rotation of back side view of the accessible surface.

The three-dimensional structure modeling generated five (5) top decoys of DehD with confidence scores (c-score) ranging from −0.52 to −2.98. The one with the lowest c-score (−0.52) represents the best model and was the DehD structure selected for this study (see supplementary material). The determined electrostatic surface potential (ESP) of DehD consists of almost equally charged pockets (47.305 × 10^5^ each). This gives an overall neutral surface as observed from different viewpoints (Figure 3). However, the surfaces at the gates of the clefts were mostly acidic (red) compared to the basic surface found inside (blue).

The difference in charges observed in the structure of DehI and DehD may be influenced by its substrate specificity. This also distinguishes DehD from the DehI enzymes, which have highly basic entrances and highly electropositive substrate binding cavities. This might explain the specificity that DehD has for D-specific substrates.

### 2.3. Structural Model Refinement

**Figure 4:**
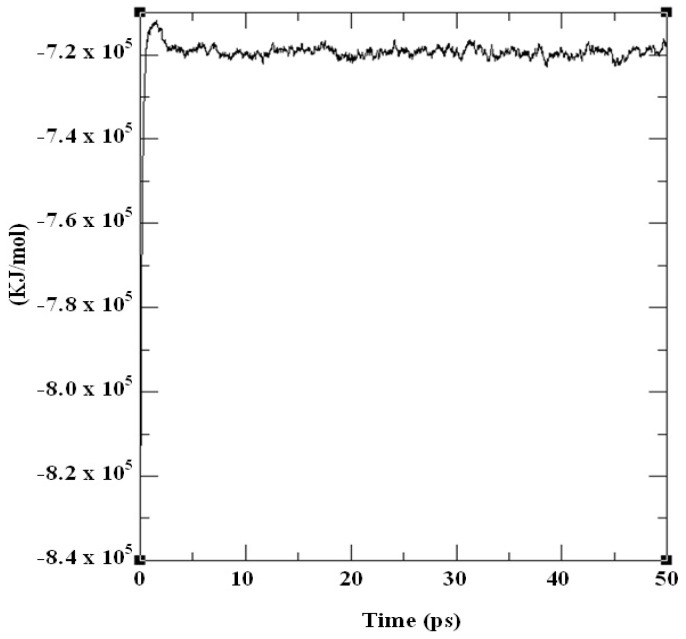
Total energy for the molecular dynamics simulation of DehD.

**Figure 5:**
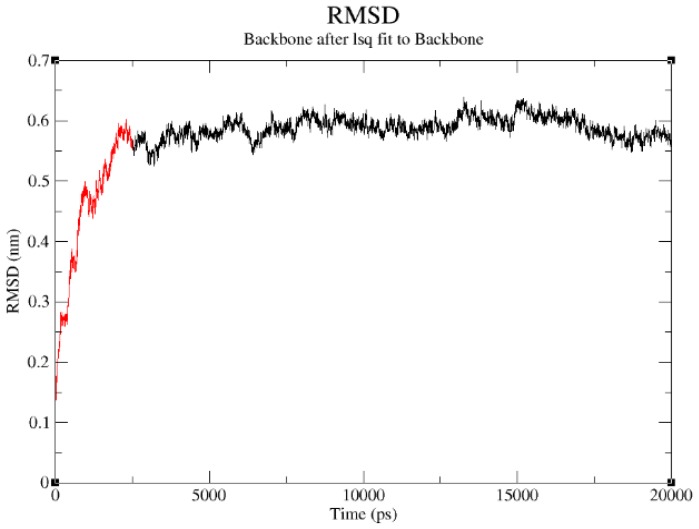
RMSD of DehD stable conformation observed after the extension of a simulation run from 5000 ps to 20,000 ps. Extension from 5000 ps to 20,000 ps shown in black color.

**Figure 6:**
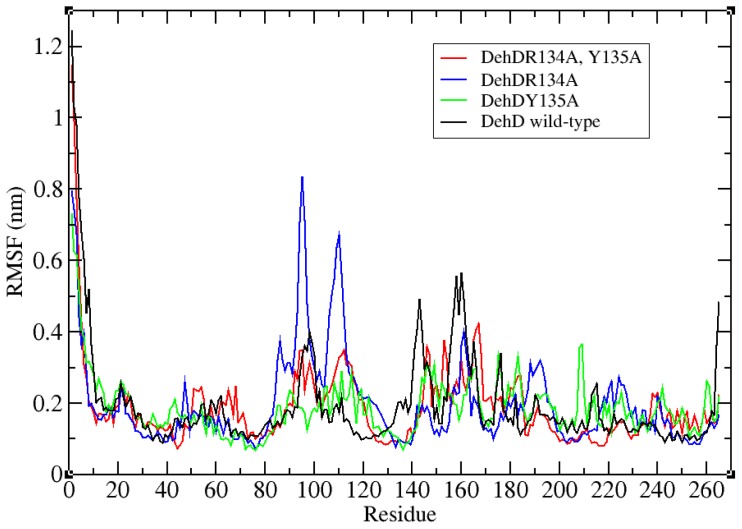
RMS fluctuations of amino acid residues of DehD compared to its variants. (**i**) black is wild-type DehD. (**ii**) blue is the R134A mutant. (**iii**) green is the Y135A mutant. (**iv**) red is the double substitution R134A/Y135A mutant.

The structural refinement was carried out using molecular dynamics simulation over the equilibration course (Figure 4) and exhibits a RMSD plot for the DehD model that flattens from 5000 ps to 20,000 ps (Figure 5). A DehD coordinate file was generated and extracted at this trajectory. At *t* = 0–5000 ps, when data were collected every 1 ps and tau of 0.1, the structure was observed to fluctuate between 0 and 0.6 nm at 6 Å and stabilize after an extension of up to 20,000 ps solvating using 173,529 gen seed at 300 K. The root mean square fluctuation (RMSF) was computed for DehD (Figure 6) and its variants and compared to a constant total energy −7.2 × 10^5^ kJ/mol of the system and an RMSD of 0.60 nm. The RMSF of the DehD amino acid residues generally gave a best-fit conformation for the three variant proteins (R134A, Y135A, and R134A/Y135A) of approximately 0.88 nm and 0.68 nm for wild type DehD. The initial model with 71.3% quality was refined by MD simulation at 5000 ps to obtain a model quality of 79.6% which was subsequently used for docking studies.

**Figure 7:**
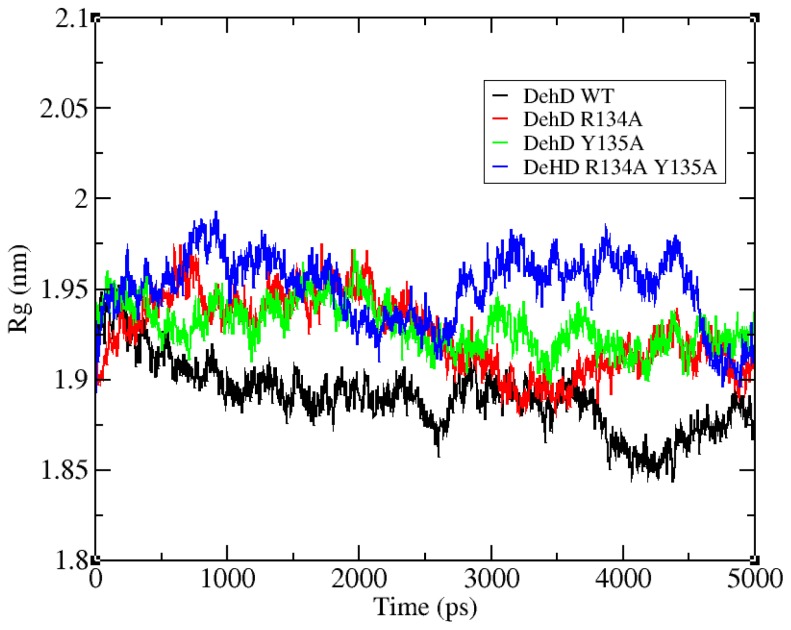
The radius of gyration of DehD compared with its variants. (**i**) black is DehD wild-type (DehD WT). (**ii**) red is the R134A mutant. (**iii**) green is the Y135A mutant. (**iv**) blue is the double substitution R134A/Y135A mutant.

The R134 and Y135 residues were found fit at 0.2 nm gyration and their corresponding mutants R134A and Y135A were found fit at 0.4 nm (Figure 6). All the residues in the tested proteins fluctuate averagely around or near wild-type values (approximately 0.2 nm). However, residues 95 and 104 in the variant R134A fluctuate at 0.88 nm and 0.68 nm, respectively, higher than for any other residue. Additionally, wild-type DehD and its variants amino acid residues were observed to fold and collapse initially within an average distance of 1.90–1.95 nm (Figure 7), with an increase in gyration over time for the variants and a decrease for the wild type. At 2600 ps, the gyration of wild-type DehD dropped to 1.85 nm and that of its variants amino acid residues dropped to 1.92 nm. DehD carrying the double substitution mutation (R134A and Y135A) unfolded and collapsed at longest gyration distance of 1.98 nm about its x, y and z-axes before attaining a stable conformation. All DehD variants stabilize at a gyration distance of about 1.92 nm at 2800–3000 ps.

### 2.4. Structural Model Validation

**Figure 8:**
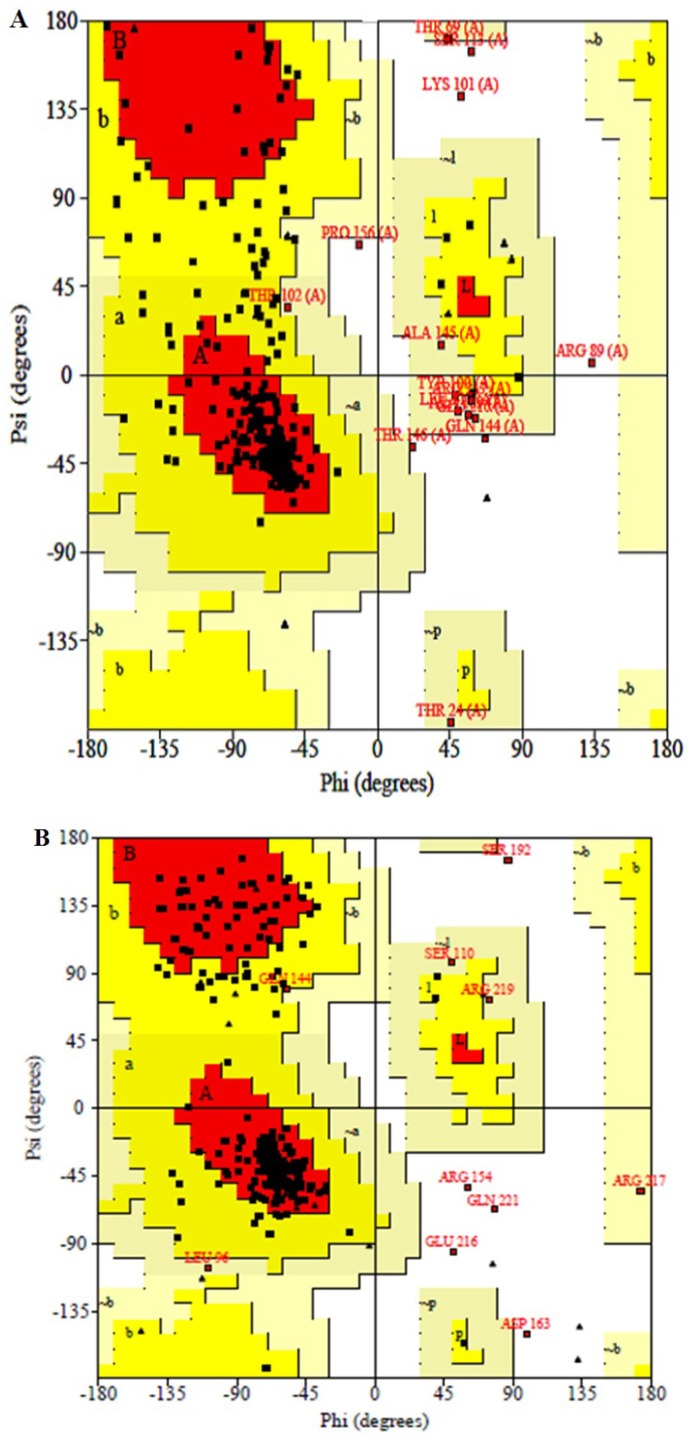
Ramachandran plot of the 3D structure of DehD evaluated by the PROCHECK program. (**A**) Model before refinement. (**B**) Model after MD simulation. The most favored and favored region are indicated with red and yellow colors, respectively. The generously allowed region is shown in pale yellow and the disallowed region is in white color.

To validate the modeled structure of DehD, Ramachandran maps [40] were drawn before as well as after MD simulation and structures analyzed using PROCHECK (a well-established program to check the stereochemical quality of protein structures) [39]. The result for the model before simulation (Figure 8A) reveals that the phi/psi angles of 71.3% of the residues in the initial model (before MD simulation) fell in the most favored regions, 22.6% in additional allowed regions, 3.9% in generously allowed regions and only 2.2% amino acid residues (Thr80, Ser113, Lys101, Arg89 and Thr146) were found in the disallowed regions. It also showed that the main chain bond length of the model is 100% within the limits, 89.4% of the main chain bond angles also within the limits and 86.4% planar groups were within the required limits. Maximum deviation of the residues was 18.5%. G-factors also indicated −0.72 dihedrals, −0.15 covalent and attain overall G-factor of −0.47. Although five amino acid residues; Thr80, Ser113, Lys101, Arg89 and Thr146 were found in the disallowed regions, they are not among the predicted active site amino acid residues to compromise the functional capability of the enzyme.

For the validation of structural model after MD simulation, Ramachandran plot [40] (Figure 8B) showed that the phi/psi angles of 79.6% are in the most favored regions only, 16.1% in additional allowed regions, 2.2% in generously allowed regions, and 2.2% amino acid residues (Arg154, Asp163, Ser192, Glu216 and Gln221) were in disallowed regions. The average G-factors were –0.65 phi/psi, −0.68 chi1–chi2 plots, −0.19 chi1 plots only, 0.49 chi3 and chi4 plots, −1.08 omega deviation, −0.63 dihedrals, 0.47 main chain bonds, −2.45 main chain angles and −0.78 overall G-factors. The bandwidths quality of the model slightly deviated from the mean, resulting in a quality of 79.6% at a stable conformation after 20 ns simulation when compared to the crystal structure of DehI from *Pseudomonas putida* strain PP3 [13]. The structural model had only 2.2% of its residues in the disallowed region and the worst position of the Omega angle standard deviation from the mean bandwidth was only 3°. All other parameters were more accurate and were found within the plot. The structural model here produced is, therefore, a presumably good model of DehD. However, crystallization of DehD is recommended to further confirm the validity of the present model. The low value of 79.6% model quality might be attributed to the fact that DehD also has very low similarity to any of the crystal structures in the protein database (PDB), and the modeling server utilized the only available template with reasonable resemblance. When the active site of DehD was superimposed on that of the template protein employed by I-TASSER Server that was used by modeling server for comparison, it was found that the two proteins had dissimilar active sites and may not have the same catalytic residues. When the model was further reevaluated using molecular dynamics simulation, it produced equilibrium geometry that indicated a quality model for DehD. When run at a constant total energy of the system of −7.2 × 10^5^ kJ/mol and RMSD of 0.60 nm, the simulation showed that the RMSF of the DehD amino acids generally gave a best-fit conformation approximately 0.18 nm. R134A and Y135A were found to fit at 0.2 nm, and their corresponding double substitution mutant R134A/Y135A was found to fit at 0.4 nm. This observation indicates that the force field used in the simulation adequately sampled the folded state at a lower free energy basin for wild-type DehD than for its variants.

When the plot statistics of stereochemistry of the three-dimensional structure of DehD was compared with typical values (Table 2) the result shows that the percentage of residues favored in the main chain was 79.6%, *versus* a typical value of 76.6%, and the number of bandwidths from the mean was 0.3. The omega angle standard deviation was 8.4, *versus* 6.0, and the number of bandwidths from the mean was 0.8. Although the structure had −1.1 bandwidths from the mean for bad contacts per 100 residues, the overall parameter value showed zero bad contacts per 100 residues. The zeta angle standard deviation was 5.6 *versus* 3.1, giving a bandwidth of 1.6 that leads to low quality. The hydrogen bond energy standard deviation was 0.8 *versus* the typical value of 0.9 and had a bandwidth of 0.2 with −0.7 bandwidths from the mean. The hydrogen bond standard deviation −0.8 as compared to typical value −0.6 inside the Ramachandran map. The overall G-factor of DehD was −0.8 *versus* the typical value of −0.6 and was positioned inside the Ramachandran plot. The distorted geometry of the protein showed that among the five amino acid residues found in the disallowed regions, only Asp163 distorted from ideal value of 1.52 Å in the main bond lengths more than 0.05 Å from the small molecule values. Distortion in main chain bond angles showed that Arg154 distorted by 13.5° above 10° from small molecule values (ideal value 111.2°). Asp163 had a distortion in bond angles ranging from 22.7° (ideal value = 88.5°) to 79.3° (ideal value = 41.5°). Ser192 distorted in the Ser192–Asp193 chain by 11.5° (ideal value = 133.2°). Distortion among the planar groups showed that NE atom of Arg154 distorted by 0.045 Å, CB atom of Asp163 distorted by 0.032 Å and CG atom of Glu216 distorted by 0.041 Å RMS distances, respectively. The distortion of planar groups in geometry is believed not to affect the functional integrity of DehD since none of these amino acid residues are in the active site or involved in catalysis. From these observations, the authors consider the structure to be novel with no sequence similarity to the crystal structure of DehI, from *Pseudomonas putida* strain PP3 [13]. Because of this and, again, that there is no crystal structure of D-specific dehalogenase to date; the authors consider this model to represent the best model available for DehD. In addition, these observations gave an impetus for the use of this model for the prediction of functional residues and their roles in the dehalogenation processes.

### 2.5. Identification of the Binding Site and Catalytic Residues Lining the Active Site of DehD

**Figure 9:**
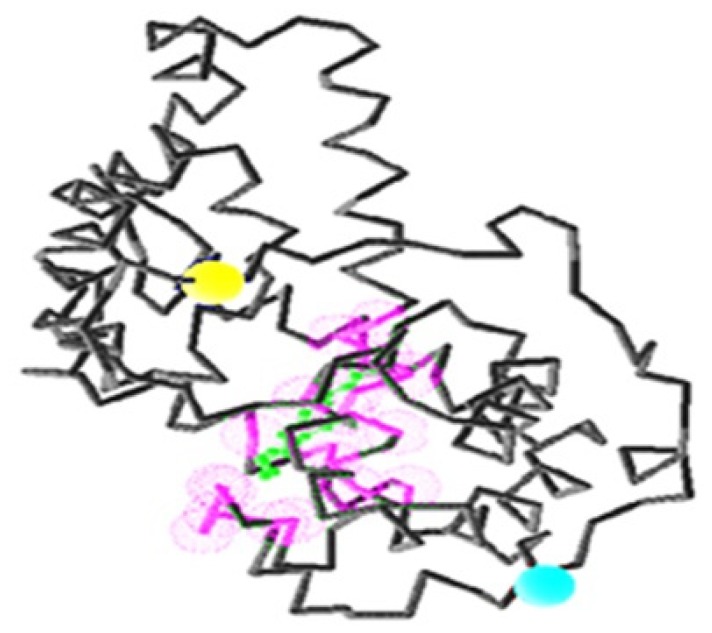
The active site of DehD. The Asp and Glu binding sites are colored blue; the Phe and Tyr binding sites are colored yellow; the Val, Ile and Leu binding sites are colored purple; the other residues (Ala, Gly, Met, Trp, Pro, Ser, Thr, Cys, Asn, Gln, Lys, Arg, and His are colored green).

**Figure 10:**
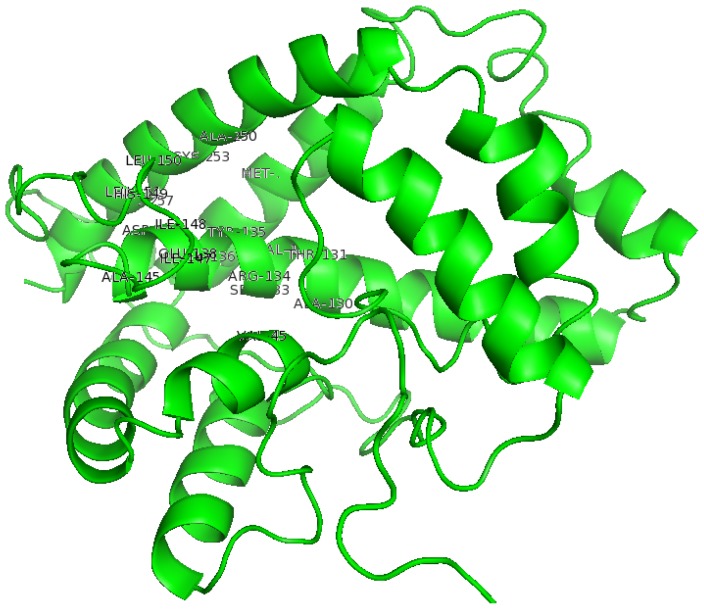
The structure of DehD showing active site amino acid residues.

**Figure 11:**
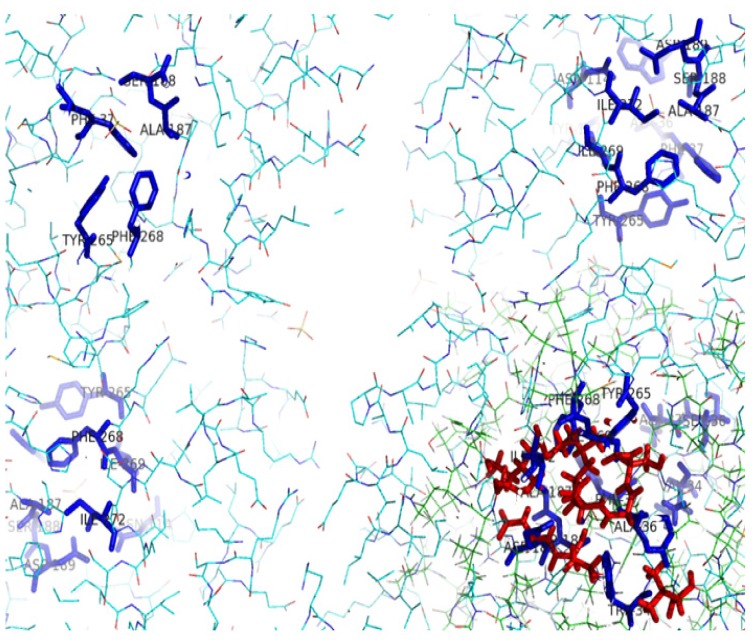
The active site of DehD superimposed onto that of DehI from *Pseudomonas putida* strain PP3. The catalytic residues of DehI from *Pseudomonas putida* strain PP3 are shown with blue sticks, and the catalytic residues of DehD with red sticks.

The results from I-TASSER revealed four possible amino acid binding sites for DehD (Figure 9): Asp and Glu binding sites; Phe and Tyr binding sites; Val, Ile and Leu binding sites; and the binding site for other residues (Ala, Gly, Met, Trp, Pro, Ser, Thr, Cys, Asn, Gln, Lys, Arg, and His). In addition, the structure revealed amino acid residues that were likely to be the catalytic residues. The amino acid residues implicated in the binding site of the DehD model were Val45, Met79, Ala130, Thr131, Val132, Ser133, Arg134, Tyr135, Leu136, Glu138, Asp139, Ala145, Ile147, Ile148, His149, Leu150, Leu151, Ala250, Cys253, and Leu257 (Figure 10). These were found to be almost centered within the structure in the buried region. When these residues were superimposed on the active site residues of the DehI (Trp34, Ala36, Phe37, Asn114, Tyr117, Ala187, Ser188, Asp189, Tyr265, Phe268, Ile269 and Ile272) from *Pseudomonas putida* strain PP3, the residues could not be aligned to each other (Figure 11). The following residues were found lining the DehD active site channel: Met79, Ala82, Thr83, Ser90, Arg134, Tyr135, Glu138, Leu245, Gln249, Ala250, Cys252 and Cys253. This observation, however, gives enough clues on the key catalytic residues and hence calls for further investigation using *in silico* mutagenesis and docking.

It was observed that the minimum constricted segment diameter of the predicted DehD active site channel was at least 0.4 Å. The electrostatic surface into this channel was highly basic compared to the rest of the molecular surface. The existence of molecular tunnels that connect one active site with another is common in enzymes with multiple catalytic sites. The apparent mechanistic advantages of molecular conduits include the protection of unstable intermediates and an improvement in catalytic efficiency due to blocking the diffusion of intermediates into the solvent [41]. It has been reported that twelve catalytic residues lining the active sites of HadD from *Pseudomonas putida* AJI were similar to the DehI group [13]. There was very low similarity between their catalytic sites, which were not superimposable. This was also observed when DehD was compared with HadD, a biochemically equivalent dehalogenase that has 15% sequence identity to DehD. There was no significant similarity in their catalytic residues. It has been reported by many researchers that it is not necessary that similar sequences should have similar catalytic residues because sequence similarity alone does not imply identity [42]. However, the highly basic electrostatic surface of DehD supports a model where in Arg134 and Glu20 are involved in the dehalogenation processes of D-2CP.

*In silico* site-directed mutagenesis has been widely used to identify the critical residues that might play a key role in catalysis. Experimentally, mutagenesis of DehD can be employed for the industrial production of chemicals, environmental remediation, pharmaceutical and medical applications [7,24,28]. The substitutions of polar residues (Arg107, Thr131, Ser133, Arg134, Tyr135, Glu138, Asp139, His149 and Cys253) with other nonpolar residues of DehD at 25 °C and 30 °C and pH 7 revealed that the DehD stability decreased at these temperatures, with the exception of the Ala substitutions and three other substitutions (S133F, R134E, H149I), which showed an increase in DehD stability. R134E and D139E had the reliability index of 7 (0 = low and 9 = high), and S133F and H149I had reliability indices of zero, Q138E and C253E had reliability indices of 6 or 7. However, all substitutions of polar residues with Ala resulted in a decrease in DehD enzyme stability, with high reliability indices of 8 and 9 for Y135A. This decrease in stability may be due to the high catalytic activities of these polar residues [43]. We therefore suggest that polar amino acid residues play a catalytic role in the dehalogenation activity of DehD.

### 2.6. Identification of the Key Catalytic Residues of DehD by Docking

**Figure 12:**
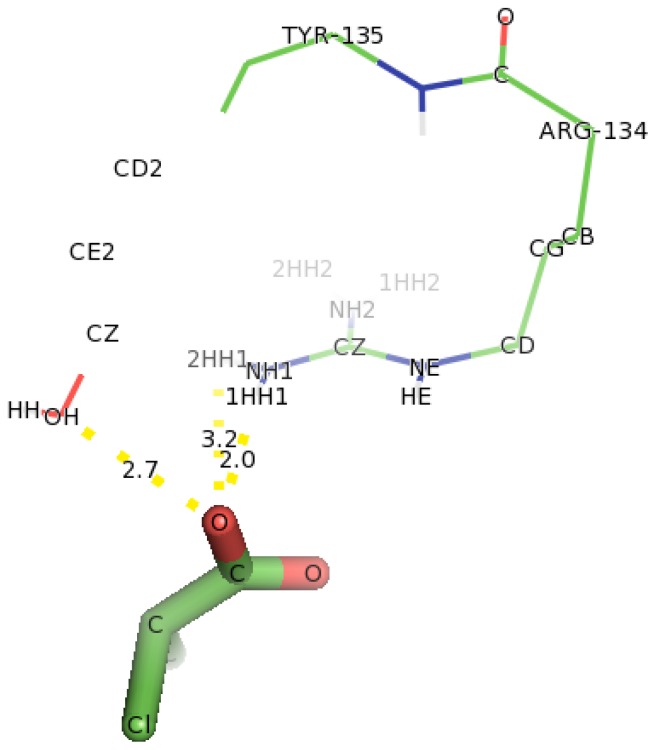
The interacting residues of DehD protein with D-2CP after 2000 ps MD simulation showing hydrogen bonding. Hydrogen bond distances are in Å.

**Figure 13:**
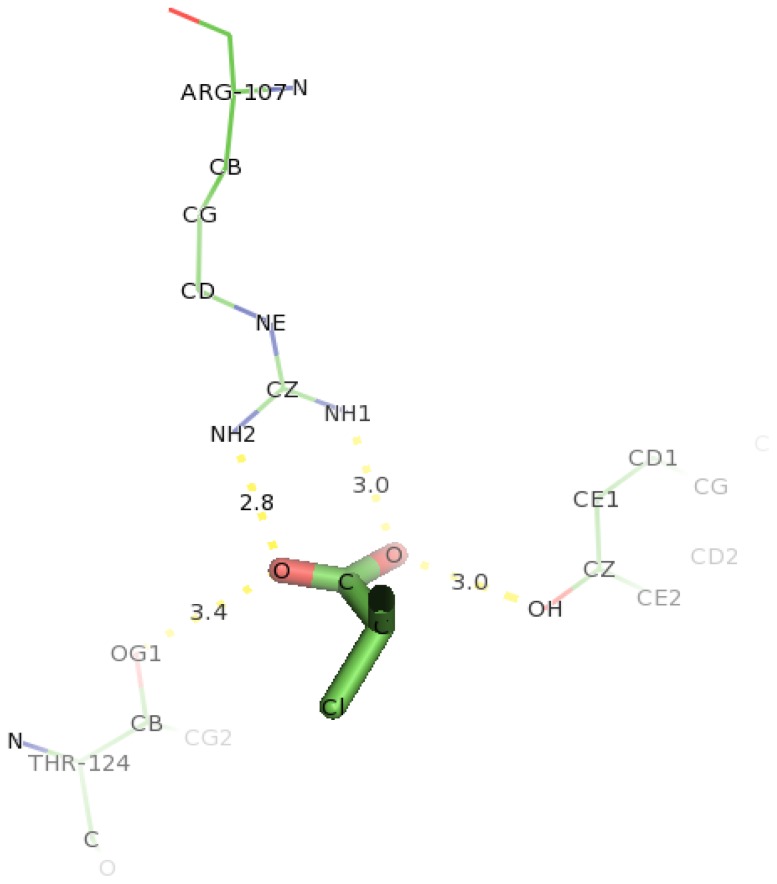
The interacting residues of DehD protein with D-2CP after 5000 ps MD simulation showing hydrogen bonding. Hydrogen bond distances are in Å.

The docking of D-2CP into the active site of DehD (Figure 12) at the energy minima that represented the best docking pose indicates the orientation of the catalytic residues. Arg134 and Tyr135 were found to interact directly with D-2CP through hydrogen bonding after 2000 ps MD simulation. Ile212 was found to be in close proximity to D-2CP, most likely stabilizing loop 9. Arg134 interacted with D-2CP by hydrogen bonding between oxygen atom of carboxylate group of D-2CP and its 2HH1 and 1HH1 atoms at distances of 3.2 Å and 2.0 Å, respectively. Similarly, Tyr135 was observed hydrogen bonding through its HH atoms of the hydroxyl group and oxygen atom of D-2CP at a distance of 2.7 Å. Ile212 was found as a close contact, possibly to stabilize the protein molecule to act on D-2CP. Kurihara and colleagues [44] have proposed that Tyr135, an aromatic polar residue, and Arg134, a basic residue, are involved in the dehalogenation process of D-2CP by indirectly attacking the halogen atom. However, docking of D-2CP in DehD after MD simulation for 5000 ps showed Arg107 interacting with D-2CP. NH1 atom of Arg107 interacted with oxygen atom of D-2CP at a distance of 3.0 Å and NH2 atom of Arg107 hydrogen-bonded to oxygen atom of the carbonyl group of D-2CP at a distance of 2.8 Å. Tyr100 hydrogen bonded to D-2CP through its hydroxyl group to oxygen atom of the carboxylate group at a distance of 3.0 Å. Similarly, Thr124 was observed in bonding to carboxylate group of D-2CP through its OG1 atom. Arg121 was observed as close contacts (Figure 13). The observed distance of hydrogen bonding and the presence of these near residues are most likely that Arg121 could be to stabilize the protein and position water molecule for hydrolytic attack of the chiral carbon. These interactions are weak considering the proposed hydrogen bond distance (short distance <2.6 Å, medium distance of 2.6–3.0 Å and long distance >3.0 Å) [45]. The presence of Arg134 as one of the key interacting residues in DehD is also in line with proposal that basic residues are in the enzyme active site and promote nucleophilic attack [46,47]. These two residues (Arg134 and Tyr135) were close to Ile212, a hydrophobic residue, which may be responsible for maintaining the stability and functional integrity of the DehD protein. This implies that these amino acid residues (Arg134 and Tyr135) are vital in maintaining DehD’s catalytic activity.

**Figure 14:**
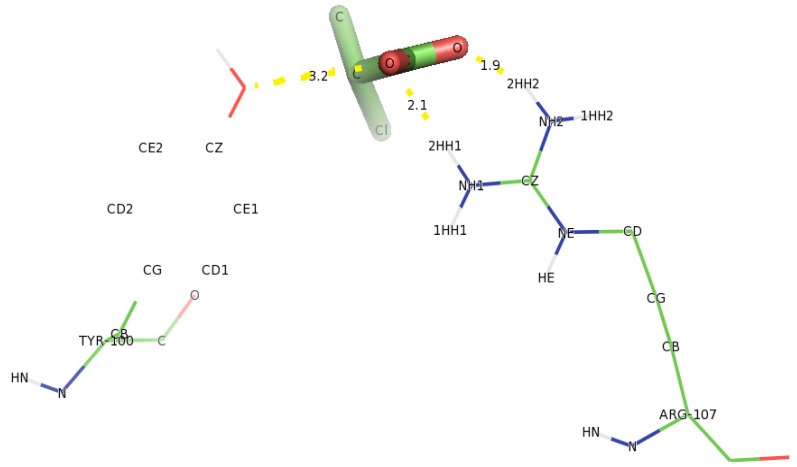
The interacting residues of DehD variant R134A with D-2CP. Hydrogen bond distances are in Å.

Analysis of docking of D-2CP into the active site of DehD variants showed that in the R134A variant (Figure 14), Tyr100 hydrogen bonded with D-2CP through the oxygen atom of the hydroxyl group of Tyr100 and oxygen atom of the carboxylate group of D-2CP at a distance of 3.2 Å. Similarly, the two hydrogen atoms (2HH1 and 2HH2 atoms) of the Arg107 side chain bonded with the oxygen atom of the carboxylate group and the oxygen atom of the carbonyl group of D-2CP at distances of 2.1 and 1.9 Å, respectively [48–52]. These distances are within the proposed distance for hydrogen bonding (short distance <2.6 Å, medium distance of 2.6–3.0 Å and long distance >3.0 Å) [45]. Arg231 poses as a close contact to aid catalysis, possibly by maintaining the correct protein conformation.

**Figure 15:**
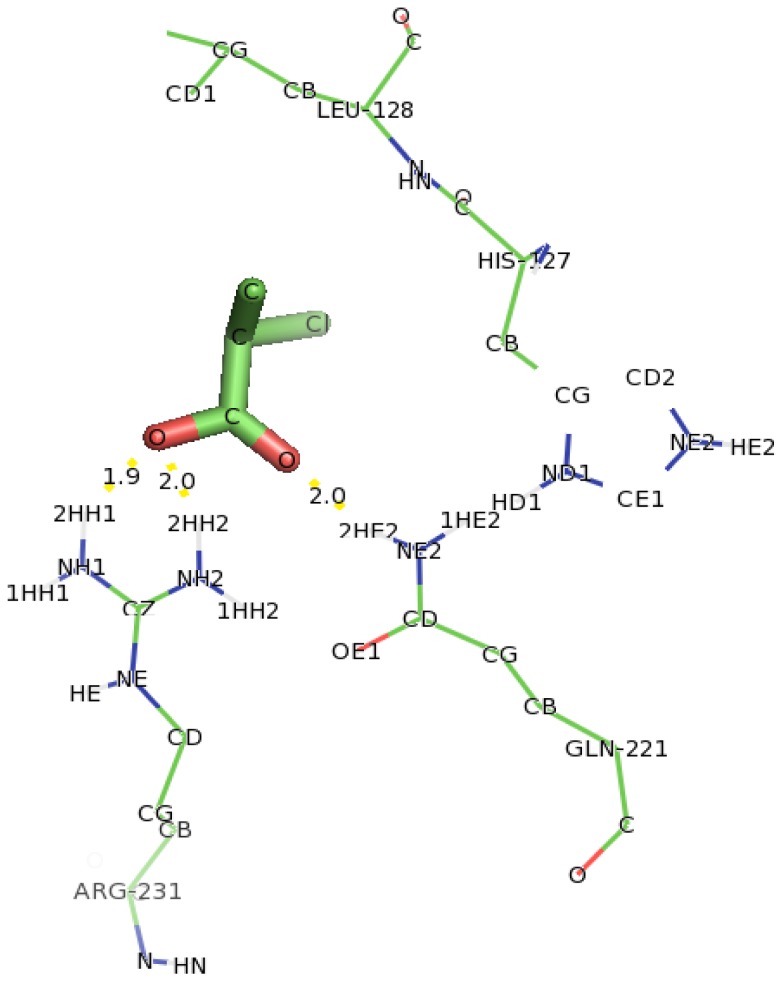
The interacting residues of DehD variant Y135A with D-2CP. Hydrogen bond distances are in Å.

In the case of Y135A variant (Figure 15), docking of D-2CP into it showed that Arg231 and Gln221 were found to interact with D-2CP. The first hydrogen atom (2HH1) of Arg231 bonded to the oxygen atom of the hydroxyl group of D-2CP at a distance of 1.9 Å, and the second hydrogen atom (2HH2) bonded to the oxygen of the carbonyl carbon in D-2CP at a distance of 2.0 Å. The second hydrogen atom (2HE2) of Gln221 bonded to the oxygen atom of the carbonyl carbon in D-2CP at a distance of 2.0 Å, both of which are within the hydrogen bonding distance [45]. Thr124, His127 and Leu128 were observed as close contacts, possibly stabilizing helix 6.

**Figure 16:**
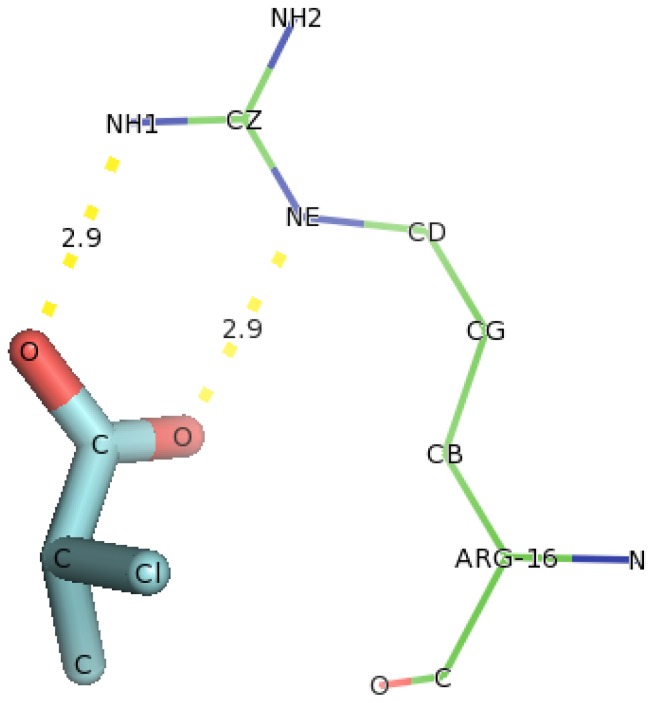
The interacting residues of DehD variant Y107A with D-2CP. Hydrogen bond distances are in Å.

Similar, docking of D-2CP into Y107A variant (Figure 16) showed only Arg16 was in hydrogen bonding with D-2CP. NE atom of Arg107 bonded to carbonyl oxygen of D-2CP and NH1 atom bonded to oxygen of carboxylate group of D-2CP both at distances of 2.9 Å. This hydrogen bond distance is also weak and no close residue to help in catalysis of D-2CP.

**Figure 17:**
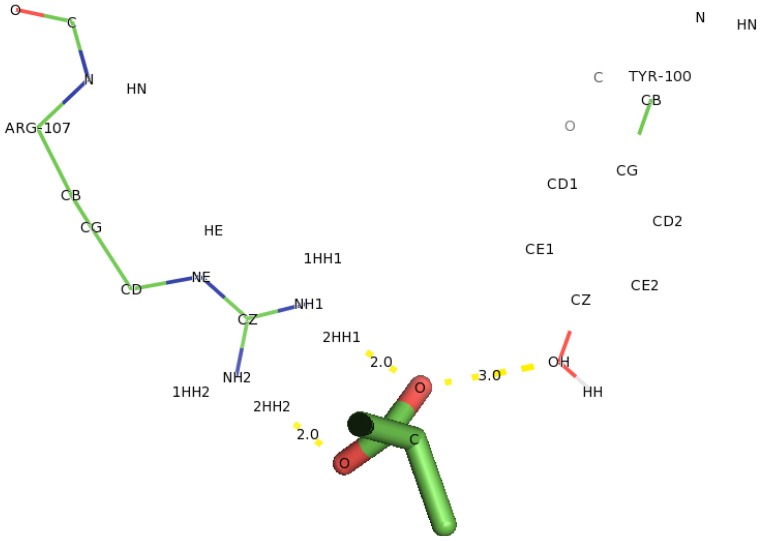
The interacting residues of the double substitution variant of DehD (R134A and Y135A) with D-2CP. Hydrogen bond distances are in Å.

Analysis of the double substitution mutants (R134A and Y135A) (Figure 17) showed Tyr100 and Arg107 in interaction with D-2CP. The oxygen atom of the phenol group of Tyr100 was observed bonding with the hydrogen atom of the carboxylate group of D-2CP at a distance of 3.0 Å. The oxygen atom of the hydroxyl group of D-2CP and the oxygen atom attached to its carbonyl carbon were hydrogen bonded with Arg107, both at distances of 2.0 Å. These interactions involving D-2CP are within the limit of the hydrogen bonding distance [45]. These observed interactions increased the binding energies of R134A and Y135A to 4.4 and 3.8 kcal/mol, respectively, suggesting that there were unfavorable changes in the binding mode of these variants. These data also confirm that Arg107, Arg134 and Tyr135 are important catalytic residues in the DehD protein.

**Figure 18:**
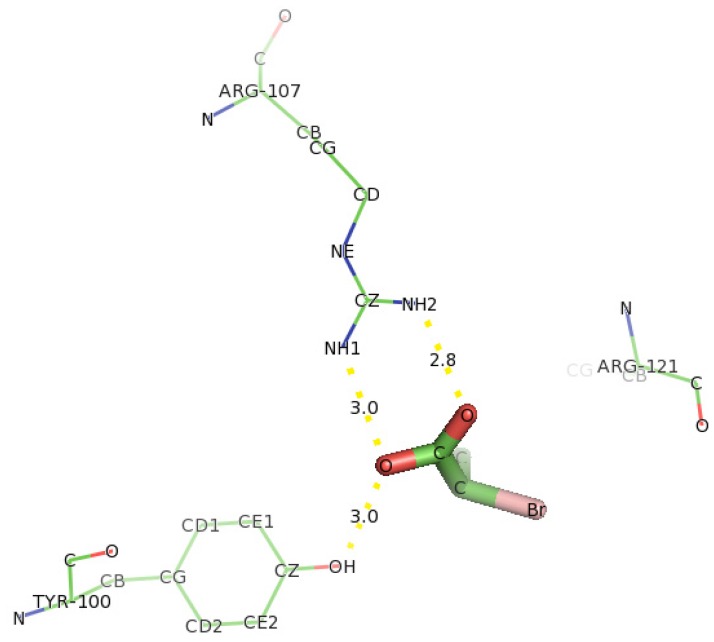
The interaction of D-2BP with catalytic amino acid residues of DehD enzyme. Hydrogen bond distances are in Å.

Docking of D-2-bromopropionate (D-2BP) into DehD (Figure 18) showed that Arg107 was found to interact with DehD through two hydrogen bondings. One hydrogen bonding was at NH1 atom of Arg107 and oxygen of carboxylate group of D-2BP at a distance of 3.0 Å and the other one hydrogen bonded at NH2 atom of Arg107 and oxygen of the carbonyl group of D-2BP at a distance 2.8 Å.

Similarly, hydroxyl group of Tyr100 was bonded to oxygen atom of carboxylate group of D-2CP at a distance of 3.0 Å. These distances are unfavorable for hydrogen bonding. Two residues (Arg121 and Thr124) were seen as close contacts for hydrogen bonding to take place between Arg107 and D-2BP. This interaction indicates Arg107 to act as key residue for DehD to act on D-2BP after 5000 ps MD simulation.

The interactions of Tyr100 and Arg107 residues with D-2CP in the single amino acid residue substitution mutants R134A and Y135A differ from those of the wild type DehD. In the wild type, after 5000 ps simulation, Arg107 interacts with D-2CP at a distance of 2.8 Å and 3.0 Å, while in R134A it interacts at distances 1.9 Å and 2.1 Å. Tyr100 interacted at a distance of 3.2 Å. There was no interaction with Tyr100 and Arg107 in Y135A and Y107A mutants with D-2CP. There was no interaction of Arg107 in wild-type DehD, but it interacted with D-2CP in the Y135A variant at distances of 2.8 Å and 3.0 Å. The difference is a result of the different folding pattern each amino acid residue imparts onto a protein when substituted. Site-directed mutagenesis of these residues (Arg100, Arg107, Arg134 and Tyr135) could be helpful in functional analysis of DehD.

**Figure 19:**
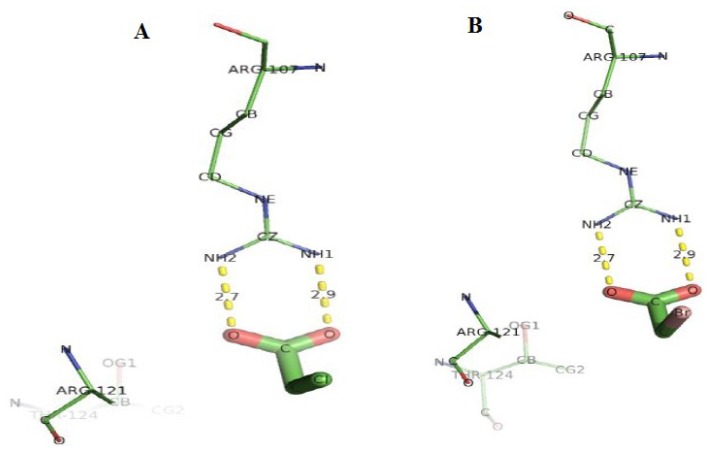
The interacting residues of DehD with (**A**) MCA (**B**) MBA. Hydrogen bond distances are in Å.

Docking of monochloroacetate (MCA) and monobromoacetate (MBA) (Figure 19) both showed Arg107 bonding with these substrates at same distances and atoms. The hydrogen bonding between both substrates and DehD were at a distance 2.9 Å from NH1 atom of Arg107 to the oxygen of the substrate and at a distance 2.7 Å from NH2 atom of Arg107 to the oxygen of the substrates. The interactions between DehD and other substrates (D-2BP, MCA and MBA) are not within the proposed hydrogen bond distances. This observation is in line with the experimental high kinetic activity (km) values for these substrates compared to D-2CP as earlier reported [29].

The docking of a water molecule into the active site of DehD (see supplementary information) revealed Arg107 and Thr124 interacting directly with a water molecule at distances 1.9 Å and 2.1 Å, respectively, after simulation for 50 ps. Similarly, docking of a water molecule after simulation of DehD for 5000 ps, attain a stable conformation and revealed that three amino acid residues, Glu20, Gly44 and Ala47, interacted with the water molecule at distances 2.2 Å, 2.4 Å and 3.0 Å, respectively. Glu20 (Asp189, with an elevated pKa of 6.6, in DehI of *Pseudomonas putida* PP3), which is negatively charged, as is Asp189, might be responsible for activating the water molecule so that it can catalyze the carbon-halogen breakdown without forming an ester intermediate [13,31]. This observation is consistent with an earlier report that Asp189 is conserved among the DehI group of dehalogenases, and that Asn114 is responsible for raising the pKa of Asp189 to enable water activation [13]. This work suggests that DehD, which is also a D-specific dehalogenase, might use a similar method, with Glu20 activating water for the nucleophilic attack on the chiral carbon of D-2CP. The role of Glu20 as an acidic residue in the present study supports earlier reports that the Asp in D-specific dehalogenases is responsible for activating the water molecule for nucleophilic attack on the chiral carbon in α-haloalkanoic acids [13,28]. Furthermore, substantial involvement of Arg134 and Tyr135 in the dehalogenation of D-haloalkanoic acid was observed. Moreover, Arg134 was the key catalytic residue of DehD and Glu20 was responsible for activating the water molecule. However, confirmation of this model using experimental procedures is of paramount significance.

### 2.7. Docking of Other Substrates into DehD Active Site

**Figure 20:**
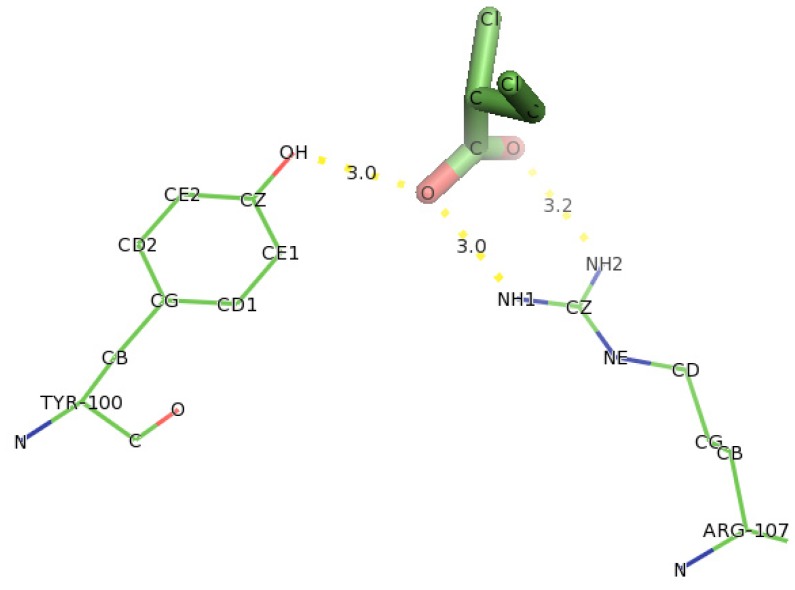
The interacting residues of DehD with D,L-2,3-DCP. Hydrogen bond distances are in Å.

In docking studies involving D,L-2,3-DCP into DehD (Figure 20), two amino acid residues (Arg107 and Thr121 were observed to interact with D,L-2,3-DCP. Although DehD was reported to act on D,L-2,3-DCP with a higher activity (km = 0.38 mM) compared to that of D-2CP (km = 0.06 mM) [29]. NH1 atom of Arg107 interacted oxygen of the carboxylate group of D,L-2,3-DCP at a distance of 3.0 Å and its NH2 hydrogen atom bonded to oxygen of the carbonyl group of DehD at a distance of 3.2 Å. Hydroxyl group of Tyr100 bonded to carboxylate oxygen at a distance of 3.0 Å. Arg121 were observed as near residues. These hydrogen bond distances are long for the protein to act on D,L-2,3-DCP. Involvement of Arg107 in processing D,L-2,3-DCP calls for experimental investigation.

**Figure 21:**
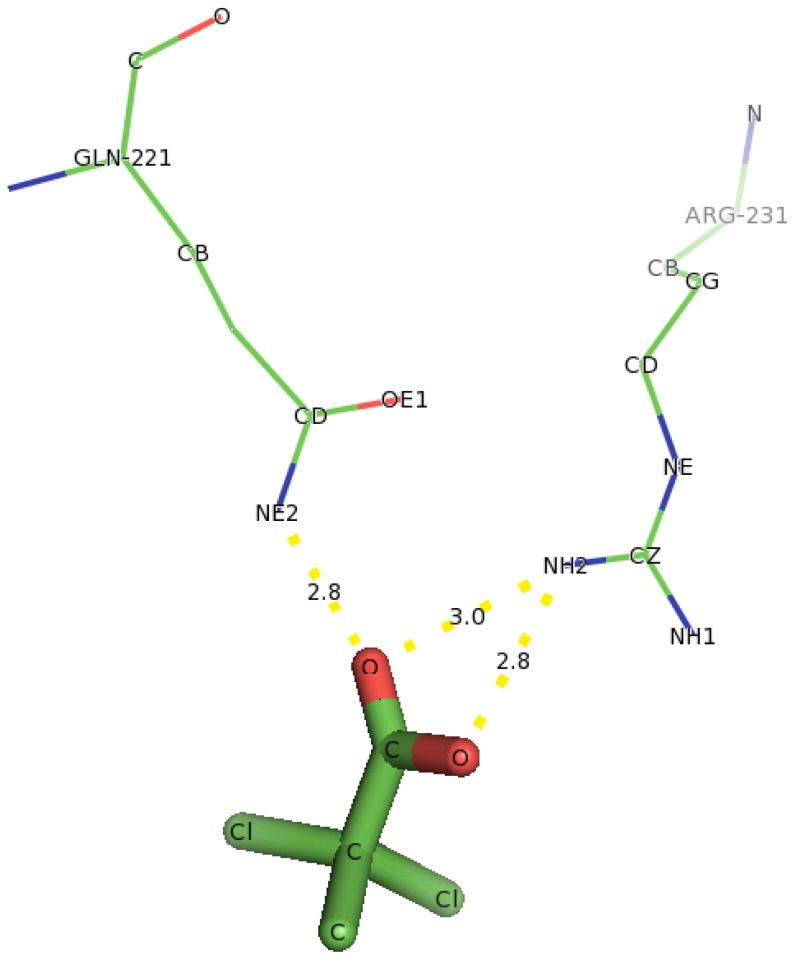
The interacting residues of DehD with 2,2-DCP. Hydrogen bond distances are in Å.

Docking of 2,2-DCP into the active site of DehD (Figure 21) also showed interaction with the residues not earlier predicted in the protein binding site. Gln221 was observed in hydrogen bonding through its NE2 atom with oxygen atom of the carbonyl group of 2,2-DCP at a distance of 2.8 Å. Arg231 bonded to oxygen atom of the carbonyl group of 2,2-DCP through its NH2 atom at a distance of 3.0 Å and also bonded to oxygen atom of the carboxylate group of 2,2-DCP at a distance of 2.8 Å. His127, Thr124, Leu129 and Thr131 were observed as close contacts. This hydrogen bond distance is also not favorable for dehalogenation process to occur. This is in line with the experimental findings that DehD does not act on 2,2-DCP [29].

Docking of 3-chloropropionate (3-CP) into the active site of DehD was carried out to investigate the assertion that it does not act on this substrate. The docking showed that there was no hydrogen bonding with any active-site residue. This observation is in tandem with the reported literatures that 3-CP, 2,2-DCP and are not substrates of DehD [26,28,29]. 3-CP, a toxic pollutant has been reported to be processed only by a strain of *Micrococcus denitrificans*, *Pseudomonas* sp. B6P, *Rhodococcus* sp. HJ1, and *Bacillus* sp. CGMCC 4196 [27,30,53,54]. This result further signifies that the model is actually a good model for DehD enzyme.

**Figure 22:**
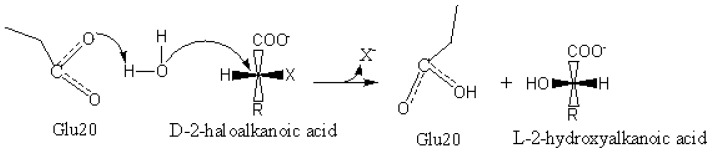
A proposed reaction mechanism for DehD, showing Glu20 activating the water molecule to attack the carbon–halogen bond and displace chloride without the formation of an ester intermediate.

It was observed from docking analysis that Glu20 interacted with D-2CP through hydrogen bonding. Therefore, it is our belief that Glu20 indirectly attacks the chiral carbon of D-2CP through an activated water molecule, thereby breaking the carbon–halogen bond and displacing chloride ion (Figure 22). This dehalogenation process is in agreement with the proposed reaction mechanism of the representative α-haloacid dehalogenases D,L-DEX and DehI, which proceeds through a nucleophilic attack of the substrate’s chiral carbon *via* water molecule activated by Glu69 and Asp194 for D,L-DEX or Glu66 and Asp189 for DehI, resulting in the inversion of the product’s configuration [13,28,53].

The DehD enzyme kinetic studies was reported to be 0.06 mM for D-2CP, 0.48 mM for D-2BP, 0.25 mM for MCA and 0.67 mM for MBA, indicating D-2CP as a better substrate for DehD [29]. The docking studies involving different substrates of DehD reveal that Arg107, Arg134 and Tyr135 interact through hydrogen bonding. The interactions of DehD with D-2BP, MCA and MBA with Arg107 were between the range of 2.6 Å and 3.0 Å (medium distance of hydrogen bonding). This observation favors Arg134 and Tyr135 as key catalytic residues for DehD to act on D-2CP, and Arg107 as key catalytic amino acid residue for DehD to act on D-2BP, MCA, MBA and D,L-2,3-DCP. Site-directed mutagenesis of Arg107, Arg134 and Tyr135 will further reveal the roles of these residues in the dehalogenation process.

## 3. Experimental Section

### 3.1. The Primary and Secondary Structure of DehD

The sequence of D-2-specific dehalogenase was retrieved from Genebank (id: CAA63793.1) at http://www.ncbi.nlm.nih.gov/protein/1103494 [26]. The amino acid composition was determined and plotted using bioedit, hosted by a Discovery Studio 2.5 (DS2.5) visualizer (Accelrys Software Inc.: San Diego, CA, USA).

Fast family and domain prediction were carried out using PSI-BLAST on EMBL-EBI site. Similarly, functional domains were predicted using the support vector machine of DOMAC by DiANNA server [55–58].

The FASTA format of the DehD from *Rhizobium* sp. RC1 and the HadD from *Pseudomonas putida* strain AJ1 were submitted for sequence alignment using Multalin on ESPRIPT [35].

Secondary structure elements of DehD were determined using a Phyre sever that employs psipred, jnet and sspro [59–62].

### 3.2. Three-Dimensional (3D) Model of DehD

The 3D structure of the DehD was modeled using an I-TASSER server [63–65]. The result generated five decoys, and the one with the lowest energy structure was selected as the best optimized 3D structure of DehD. DehD was subjected to five iterations of energy minimization in the DS2.5 suite (Accelrys Software Inc.: San Diego, CA, USA), further subjected to MD simulation and then visualized using PyMol [66].

### 3.3. Structure Refinement and Validation

DehD was subjected to molecular dynamics simulation using a parallel version of GROMACS 4.5.1 on Linux to determine the structural integrity of the model [67]. Wild-type DehD and its variants were treated for refinement in “space water” molecules, a simple point charged–extended water molecule consisting of three atomic sites, rigid geometry, and a tetrahedral bond angle [68]. The box edge was set at approximately 1.0 nm, and centered at 4.19 Å. After solvation, the system was neutralized according to the Ewald equation (which describes the long-range electrostatics) in order to control the force field and the conditions for molecular dynamics, such as the molecular dynamics parameter (mdp). The file was prepared to set GROMACS for energy minimization and equilibration of the system. To allow the water molecules to move into the protein during equilibration, atomic positions were restrained for 50 ps. To compute long-range electrostatics, Coulomb potentials were treated using “Particle Mesh Ewald” (PME) electrostatics [69–70]. A linear constraint algorithm was used to fix all bond lengths in the system [71]. Water molecules were checked for equilibration prior to the production of protein for simulation in “ionwet” and “invacuo.” MD simulation was performed in the NVT ensemble with number of particles; volume of the system and temperature of 300 K were kept constant. After simulation, the time evolving coordinates of the system (trajectories) were processed and analyzed (see supplementary material). The root mean square distance (RMSD) was compared to the crystal structure of *Pseudomonas putida* strain PP3 [13], and the root mean square fluctuation (RMSF) of atom position was generated. The stereochemical quality of the model was determined using PROCHECK hosted by SWISS-MODEL [39,72].

### 3.4. Identification of Catalytic Residues and Substrate Docking into the Active Site of DehD

The key catalytic residues of proteins that might be responsible for binding to a ligand can be investigated using site-directed mutagenesis. It has been established that detrimental mutations in structural proteins trigger changes in structural features such as altering the surface charge allocation and disrupting the protein packing in its core region leading to possibility of rapid genetic assortments with multiple variants. Similarly, it is expected that mutation of the catalytic residues may cause variations in its function [73]. Ala substitutions produce neutral mutations in polypeptides. Ala scanning mutagenesis removes all side chain atoms and does not introduce unusual backbone dihedral angle preferences without changing the protein orientation. This is compared to mutagenesis of Gly, which has more conformational flexibility that allows it to reside in turns within the structure, while Pro is too rigid. Pro is rarely found in enzyme active sites. Rather, it is involved in forcing sharp turns in protein sequences, thereby changing the directions of the backbone and in introducing kinks into α-helices. The reason for the peculiar properties of Pro is because its side chain carbon is bound to the nitrogen of the amino group. Therefore, Pro is not recommended for mutagenesis [55]. Its electrostatic potential and steric properties are also low [56]. Ala substitution had been routinely adopted in mutagenesis studies for assessing the contribution of charged residues on the protein surface without disrupting the folding of the protein core [57].

An I-TASSER server [63–65] was used to model the structure of DehD which was simulated at 5000 ps and finally used to predict possible substrate binding sites and catalytic residues in DehD. The structural models of D-2-chloropropionate (D-2CP), D-2-bromopropionate (D-2BP), monochloroacetate (MCA), monobromoacetate (MBA), 2,2-dichloropropionate (2,2-DCP), D,L-2,3-dichloropropionate (D,L-2,3-DCP), 3-chloropropionate (3-CP) substrates and the water molecules used for the docking studies were extracted from the PUBCHEM server http://pubchem.ncbi.nlm.nih.gov [74]. The PDB structure files were edited by adding and merging nonpolar hydrogen into the system. Kolmann and Gasteiger charges were added to the system and torsion angles were assigned using ADT. Docking parameter files were generated and converted to the “PDBQT” file format for docking [75]. All molecular docking were carried out using AutoDock Tools version 4.2 [50]. Receptor PDB files were loaded into the grid box. The grid box was set to encompass the entire DehD protein or its variants by adjusting the thumbwheel to 1 Å and the dimension wheel to 26, 40 and 28, yielding total grid points of 68,921. Adjustments for the DehD variants were made until they fit at the center of the box to obtain the correct ligand pose in the best-ranked position with the lowest binding energy. The DehD coordinate file was edited by removing the water molecules, adding hydrogen and merging nonpolar hydrogen. Kolmann and Gasteiger charges were added on to the structure. Autoligand was allowed for 10 runs to obtain favorable local minima. The active site residues were specified. Ligands (D-2CP, D-2BP, MCA, MBA, 2,2-DCP, D,L-2,3-DCP, 3-CP and water molecule) were also prepared in a similar manner as receptors (DehD and its variants). Torsion angles and their roots assigned to ligands. Rigid docking parameter of 100 runs and 250,000 evals was generated. Docking was reclustered for three tolerances (0.5, 1.0 and 2.0). The clustering tolerance with the largest conformations at RMS of 1.0 nm was selected and complexes written, saved in pdbqt files for visualization of key catalytic residues using PyMol [39,76]. *In silico* site-mutagenesis was performed by SNAP [77], and the mutant stability determined using I-Mutant [78] to computationally access the effect of amino acid substitution in the DehD molecule.

## 4. Conclusions

With the aim of identifying the catalytic residues of DehD, the researchers present the first model of DehD generated by the *ab initio* technique. The structure was found to consist of α-helices and loops. The predicted catalytic residues were found to be buried. Furthermore, it was predicted that site-directed mutagenesis of the catalytic residues of DehD would also reveal their involvement in the dehalogenation of D-2CP. Sequence alignment of DehD with HadD indicates low identity (15%) between these two proteins. This low sequence similarity suggests that the catalytic residues of these proteins may not share the same catalytic activity.

Arg134 is conserved among similar dehalogenases and was therefore hypothesized to play a key role in the dehalogenation process. Arg107, Arg134 and Tyr135 were found to interact with the substrates and are assumed to be the catalytic residues of DehD that are involved in the dehalogenation of D-2CP and D-2BP, while Glu20 is predicted to activate the water molecule that attacks the carbon halogen bond on the α-carbon, thereby releasing chloride ion. A similar docking of 3-CP into the active site of DehD did not predict the substrate to be in the enzyme “hot spot,” and no hydrogen bonding was observed. Hence, we conclude that 3-CP is not a potential substrate for DehD. This confirms that the recalcitrance of the toxic pollutant D-2CP is an indication that its dehalogenation is not simply a spontaneous hydrolytic process but requires the intervention of dehalogenating enzymes. Therefore, it is our belief that experimental site-directed mutagenesis of DehD focusing on the catalytic residues could unveil their catalytic roles in the haloalkanoic acid dehalogenation processes. It is our strong belief that the mutants that thus generated could be used in bioremediation, sewage treatment, manufacturing and industrial processes.

## Supplementary Information



## Figures and Tables

**Table 1:** Number and lengths of loops and helices in DehD.

Number of amino acid residues in the loop	Loop length	Number of amino acid residues in the helix	Helix length
24	M1–T24	33	G5–A37
5	F38–W42	8	M43–I50
2	P51–I52	16	S53–G68
1	T69	18	R70–V87
14	E88–K101	9	T102–S110
3	G111–S113	28	E114–R141
25	G142–G166	3	T167–G169
24	F170–D193	17	E194–I210
9	L211–R219	4	G220–I223
4	S224–V227	37	G228–L264
1	P265		
Total (percentage)	112 (42.26%)		173 (65.28%)

**Table 2:** Comparison of the stereochemical properties of DehD to typical values.

Stereochemical parameter	Parameter value	Typical value	Bandwidth	Number of bandwidths from mean
Percentage residues in favored region	79.6	76.6	10.0	0.3
Omega angle standard deviation (degrees)	8.4	6.0	3.0	0.8
Number of bad contacts/100 residues	0.0	10.5	10.0	−1.1
Zeta angle standard deviation (degrees)	5.6	3.1	1.6	1.6
Hydrogen bond standard deviation	0.8	0.9	0.2	−0.7
Overall G-factor	−0.8	−0.6	0.3	−0.6
