# Supplementary material for: Structure Prediction, Molecular Dynamics Simulation and Docking Studies of D-Specific Dehalogenase from Rhizobium sp. RC1
Source: Int J Mol Sci. 2012 Nov 26;13(12):15724–54. doi: 10.3390/ijms131215724 (PMC3546658; doi:10.3390/ijms131215724)
Supplement: Supplementary file 1 [file ijms-13-15724-s001.pdf]

Supplementary Information

Figure S1. Amino acid sequence alignment of DehD and HadD. An alignment with the consensus greater than 50% is shown in red blocks with white characters. The interacting residues of DehD are shown by black stars.

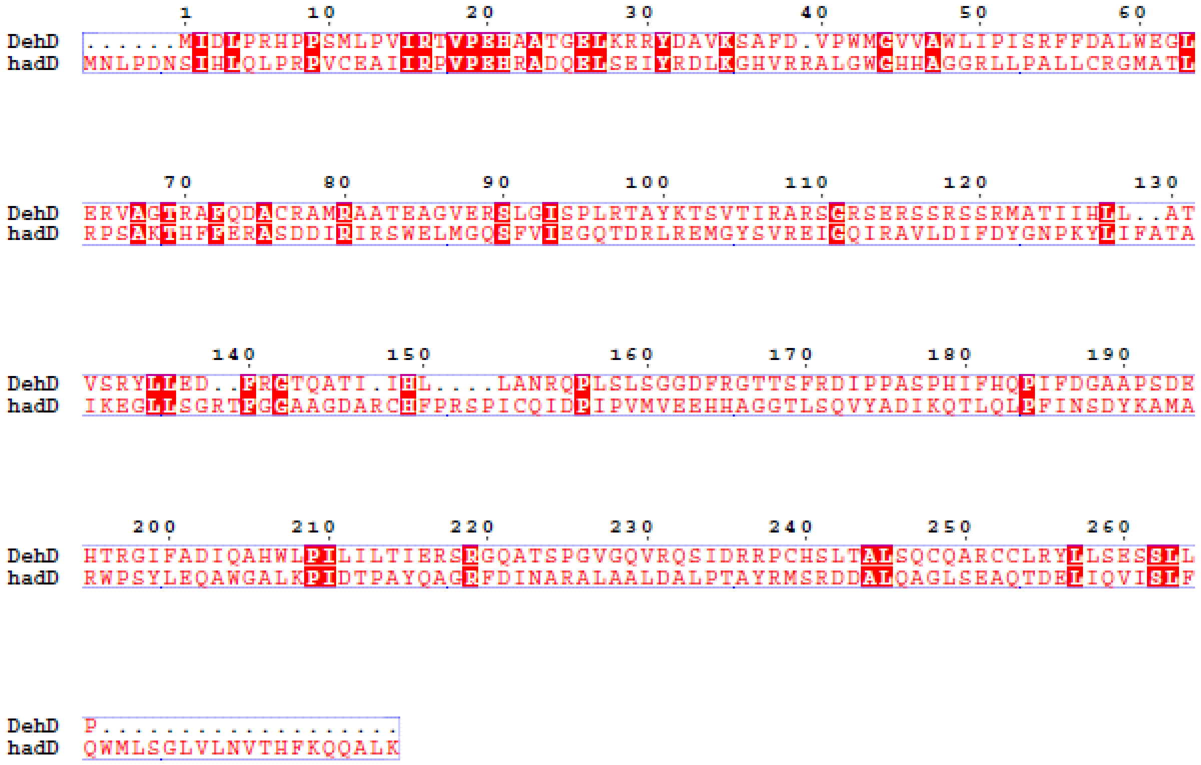

Data S1. em.mdp file for Molecular dynamic simulation.

```
define          = -DFLEXIBLE
constraints     = none
integrator      = steep
dt              = 0.002    ; ps!
nsteps          = 400
nstlist         = 10
ns_type         = grid
rlist           = 1.0
coulombtype     = PME
rcoulomb        = 1.0
vdwtype         = cut-off
rvdw            = 1.4
optimize_fft    = yes;   ;Energy minimizing stuff;
Emtol           = 1000.0
Emstep          = 0.01
```

Date S2. pr.mdp file for Molecular dynamic simulation.

```
define             = -DPOSRES
constraints        = all bonds
Integrator         = md
dt                 = 0.002          ; ps!
nsteps             = 25000          ; total 50.0 ps
nstcomm            = 10
nstxout            = 250            ; collect data every 0.5 ps
nstvout            = 1000
nstfout            = 0
nstlog             = 10
nstenergy          = 10
nstlist            = 10
ns_type            = grid
rlist              = 1.0
coulombtype        = PME
rcoulomb           = 1.0
vdwtype            = cut-off
rvdw               = 1.4
pme_order          = 4     ; Use 6, 8 or 10 when running in parallel
ewald_rtol         = 1e-5
optimize_fft       = yes
DispCorr           = no     ; Berendsen temperature coupling is on
Tcoupl             = v-rescale
taut_t             = 0.1         0.1
tc-grps            = protein      non-protein
ref_t              = 300      300    ; Pressure coupling is on
Pcoupl             = parrinello-rahman
Pcoupltype         = isotropic
tau_p              = 0.5
compressibility  =4.5e-5
ref_p              = 1.0      ; Generate velecities is on at 300 K.
gen_vel            = yes
gen_temp           = 300.0
gen_seed           = 173529
```

Date S3. md.mdp file for Molecular dynamic simulation.

```
constraints      = all-bondsIntegrator        = md
dt               = 0.002           ; ps!
nsteps           = 500000          ; total 1000 ps
nstcomm          = 10
nstxout          = 500             ; collect data every 1 ps
nstvout          = 0
nstfout          = 0
nstlist          =10
ns_type          = grid
rlist            = 1.0
coulombtype      = PME
rcoulomb         = 1.0
vdwtype          = cut-off
rvdw             = 1.4
pme_order        = 4         ; Use 6, 8 or 10 when running in parallel
ewald_rtol       = 1e-5
optimize_fft     = yes
```

Date S3. *Cont.*

```
DispCorr         = no       ; Berendsen temperature coupling is on
Tcoupl           = v-rescale
taut_t           = 0.1            0.1
tc-grps          = protein        non-protein
ref_t            = 300            300      ; Pressure coupling is on
Pcoupl           = parrinello-rahman
Pcoupltype       = isotropic
tau_p            = 0.5
compressibility  =4.5e-5
ref_p            = 1.0           ; Generate velecities is on at 300 K.
gen_vel          = yes
gen_temp         = 300.0
gen_seed         = 173529
```

© 2012 by the authors; licensee MDPI, Basel, Switzerland. This article is an open access article distributed under the terms and conditions of the Creative Commons Attribution license (http://creativecommons.org/licenses/by/3.0/).
